# Environmental Pawprint of Dogs as a Contributor to Climate Change

**DOI:** 10.3390/ani15213152

**Published:** 2025-10-30

**Authors:** Antonina Krawczyk, Bożena Nowakowicz-Dębek, Anna Chmielowiec-Korzeniowska, Hanna Bis-Wencel

**Affiliations:** Department of Animal Hygiene and Environmental Hazards, Faculty of Animal Sciences and Bioeconomy, University of Life Sciences in Lublin, 20-950 Lublin, Poland; bozena.nowakowicz@up.lublin.pl (B.N.-D.);

**Keywords:** dog waste, nutrient emissions, composting, greenhouse gases, companion animals, urban biodiversity, sustainable pet care

## Abstract

**Simple Summary:**

Dogs are common human companions, but their environmental impact is rarely assessed. While the emissions and waste of farm animals have been extensively studied, dogs are usually regarded as individual pets rather than as a large population that collectively influences the environment. Dog feces and urine release nitrogen, phosphorus, and organic matter into soil and water, while daily care generates plastic waste from food packaging, toys, and waste bags. In urban areas with high dog densities, these emissions can reach levels comparable to those from small-scale livestock farms. Owners’ decisions—such as diet composition, choice of food packaging, and methods of waste collection—play a key role in reducing this environmental footprint. Providing nutritionally balanced diets, using sustainable packaging, and implementing safe waste treatment methods such as composting can help mitigate emissions. Furthermore, raising public awareness through owner education and local policies could lead to meaningful environmental improvements. More comprehensive research on the environmental pawprint of dogs, along with harmonized legal frameworks that include dogs in national and international emission inventories, is essential to obtain more accurate estimates and to develop effective mitigation strategies.

**Abstract:**

The environmental impact of companion animals has received little scientific attention compared to that of livestock, even though the global dog population is rapidly increasing, particularly in urban areas. This review addresses the overlooked contribution of dogs to environmental emissions, focusing on feces, urine, packaging waste, and other care-related by-products. The current knowledge from livestock research provides useful analogies for understanding nutrient excretion and gaseous emissions from dog feces, and data on nitrogen and phosphorus inputs highlight their potential to pollute soil and water. We also examine the role of plastic waste from food packaging, waste bags, and accessories, which can degrade into microplastics, and discuss recent developments in biodegradable materials. Evidence shows that owner choices—such as diet composition, protein sources, and product selection—directly affect the environmental pawprint of dogs. Mitigation strategies include optimizing diets to reduce nutrient excretion, applying feed additives developed for livestock, and improving waste management through composting or the use of emission-reducing amendments. In conclusion, dogs should no longer be viewed merely as individual household companions but as a population with a measurable environmental pawprint. Including dogs in emission reporting systems would provide a more accurate basis for mitigation policies and sustainable urban planning.

## 1. Introduction

The domestication of dogs and their complete dependence on human care entails responsibility not only for their health but also for the environmental consequences [1], particularly as ecological awareness regarding animal husbandry has increased in recent years. Much attention has been devoted to the pressure exerted by livestock farming and its associated greenhouse gas (GHG) emissions. By contrast, the ecological footprint left by companion animals is rarely considered, despite the fact that their presence constitutes a significant environmental burden.

Today, it is estimated that nearly one billion dogs live worldwide, including both owned and free-roaming animals. According to Cowan et al. [2], the global population of companion animals includes approximately 700 million dogs and 900 million cats. This scale is not accounted for in any existing estimates of emissions or environmental burdens related to waste production. The scale of this issue is further illustrated by the size of the pet food, accessories, and veterinary services market, which exceeded USD 200 billion in 2021 [3]. Within the European Union alone, the European Pet Food Industry Federation (FEDIAF) reports that nearly 10 million tons of dog and cat food are produced annually. Per capita, consumption levels are highest for dogs, averaging 37.3 kg per year [4].

In the United States, it has been calculated that the energy demand of dogs and cats represents nearly 20% of human caloric consumption [5]. Diets based primarily on meat and animal-derived products impose an additional environmental burden. Poultry and beef are the most common protein sources in commercially available pet food. The amount of meat required to meet the nutritional needs of dogs can be comparable to that of a human diet—even though humans, despite having greater body mass, consume more varied and largely plant-based foods. This relationship often goes unnoticed by pet owners, as dogs are not commonly perceived as emitters or consumers of natural resources. However, their meat-heavy diet generates a significant environmental pawprint, largely due to the absence of plant-based substitutes [6].

In the United States, it has been estimated that dogs and cats together consume as much as 33% of all animal-derived energy consumed by humans. On a national scale, this means they account for nearly one-third of the environmental impact of livestock production, including land, water, and resource use, as well as GHG emissions. These animals also produce more than 5 million tons of feces annually—equivalent to almost 30% of the total human fecal output in the United States [5]. Based on a life cycle assessment (LCA) performed by Yavor et al. [7], dog ownership was found to account for roughly 7% of the annual carbon footprint of an average citizen in the European Union.

Therefore, the aim of this review is to critically assess the environmental pawprint of dogs, with a particular focus on emissions and nutrient loads arising from their excreta, as well as associated waste streams. We summarize the current state of knowledge on gaseous emissions, nitrogen and phosphorus inputs, and waste management issues while also highlighting knowledge gaps and methodological challenges. In addition, we discuss available and potential mitigation strategies, drawing on analogies from livestock research where applicable. By drawing attention to this overlooked source of environmental impact, we seek to emphasize the need for further research, mitigation strategies, and the inclusion of dogs in broader environmental reporting systems. Ultimately, this work aims to raise awareness among both the scientific community and society at large, stressing that the cumulative environmental pawprint of dogs should no longer be disregarded.

## 2. Materials and Methods

In this review, information from journal articles, books, and reports was thoroughly collected and analyzed. The available literature was searched in the PubMed, Web of Science, ScienceDirect, Google Scholar, and Scopus databases using the following keywords: dogs, companion animals, pets, dog waste, dog feces, dog excrements, fertilizers, diet, nutrients, ecological pawprint, emission, peri-urban ecosystems, urban ecosystems, soil pollution, water pollution, environmental pollution, air quality, compost, compost quality, composting, plastic bags, biodegradability, nitrogen, phosphorus, ammonia, nitrous dioxide, VOCs, NO_x_, monogastric animals, and ruminants. The final database access was performed in October 2025.

Titles and abstracts of all retrieved articles were initially screened to assess their relevance to the topic. Final inclusion was based on the following criteria:

(1) Composition of dog feces and dietary factors affecting their properties;
(2) Fertilizer potential of dog feces;
(3) Environmental impact of dog feces;
(4) Overlooked emission pathways in companion animal systems, including packaging, plastics, and hygiene products;
(5) Strategies for reducing emissions from dog excreta.

The methodology for structuring this review followed the framework proposed by Arksey and O’Malley [8]. Publications considered were limited to the period of 2001–2025. Studies available only as abstracts or lacking peer review were excluded. In total, 56 scientific publications were included in this manuscript. Statistical data and information regarding household practices were further supplemented with industry reports, official documents, and credible online sources (e.g., institutional and environmental organization websites).

## 3. Results

### 3.1. Composition of Dog Feces and Dietary Factors Affecting Their Properties

Diet plays a key role in determining the composition and quality of dog feces [9,10]. Dog feces consist of undigested food residues, water, exfoliated intestinal epithelial cells, digestive secretions, and microorganisms [11]. Their chemical composition varies considerably, primarily depending on diet. In studies on the composting of dog feces, the average content of total organic carbon (TOC) was 43.5 ± 1.9% of dry matter, 4.96 ± 0.09% of total nitrogen, and 8322 ± 70 mg kg^−1^ of water-soluble polyphenolic compounds [12]. Other research conducted on female beagles showed that higher dietary protein levels increased fecal carbon and nitrogen content while reducing the C:N ratio. No significant age-related differences were found. Dog feces also contained high phosphorus concentrations—exceeding those observed in livestock kept under intensive production systems—with phosphorus levels rising as the dietary protein supply decreased [13].

Elevated protein intake may result in greater excretion of biogenic compounds in both urine and feces. According to de Frenne [14], the mean nitrogen concentration in dog urine is 18.7 g L^−1^, and that of phosphorus is 484.6 mg L^−1^. In feces, nitrogen reaches 44.3 mg g^−1^ DM and phosphorus 32.0 mg g^−1^ DM, values comparable to those found in ruminants [15]. Assuming an average daily fecal mass of 100 g [16] and urine volume of about 180 mL, it is important to consider the biogenic load entering the environment through these excreta.

Domestic dogs are facultative carnivores. Although their physiology is adapted to a meat-based diet, they can digest plant-derived ingredients due to relatively high pancreatic α-amylase activity. This adaptation, developed during domestication, enables dogs to utilize starch, which obligate carnivores such as wolves cannot digest efficiently [17]. A diet composed exclusively of meat can lead to impaired physiological functions, gastrointestinal and urinary disorders, micronutrient deficiencies, and dental problems.

An unbalanced diet, along with excessive fat intake, contributes to obesity in companion animals [18]. Fat deserves particular attention in the context of emissions from dog feces. Marx et al. [19] found that dietary fat is nearly 100% digestible by dogs, regardless of the quantity consumed per meal. The inclusion of dietary fiber enhances nutrient digestibility, intestinal motility, fecal bulk formation, and the composition of the colonic microbiota [20]. Improved digestibility of pet food components reduces the amount of biogenic substances—mainly nitrogen and phosphorus—released into the environment.

### 3.2. The Fertilizer Potential of Dog Feces and Challenges of Composting

Manure, defined as a mixture of livestock excreta and bedding material (most often straw), is a rich source of nitrogen (N), phosphorus (P), potassium (K), and sulfur (S). Soil microorganisms utilize the organic and inorganic compounds present in manure, leading to their degradation and mineralization. As a result, simpler mineral forms, such as nitrates and phosphates, become readily available for plant uptake. Manure also improves soil structure, enhancing both aeration and water-holding capacity [21]. For these reasons, sustainable methods for its utilization and the mitigation of its negative environmental effects have been developed [22].

Wuthisuthimethavee et al. [23] highlighted the potential for composting dog and cat excreta. Dog and cat feces contain higher nutrient concentrations compared with livestock manure, which can translate into improved plant growth. The authors emphasized that such material may serve as a valuable fertilizer, provided that the composting process ensures the sanitary safety of the final product.

An important factor determining composting efficiency is the carbon-to-nitrogen ratio (C:N). Livestock manure typically has a higher C:N ratio than dog feces due to the carbohydrate-rich diets of farm animals. For example, pig manure can reach a C:N ratio of nearly 30:1 [24]. A C:N ratio in the range of 25–30:1 is considered optimal for effective composting, as it minimizes nitrogen losses [25]. By contrast, dog feces are characterized by a much lower C:N ratio—approximately 8:1 [26]. Such nitrogen-rich material, without additional carbon supplementation, can hinder the composting process, leading to excessive ammonia (NH_3_) emissions and disruption of the compost’s microbial balance [27]. Therefore, effective composting of dog feces requires the addition of carbon-rich materials such as sawdust, straw, or dry leaves.

Another major concern related to the use of dog feces as fertilizer is the presence of pathogens. Direct contact poses a zoonotic risk due to the frequent occurrence of species such as Toxocara canis, Campylobacter jejuni, and Echinococcus granulosus [28]. Composting, however, provides an effective treatment pathway for organic waste, as temperatures above 55 °C are typically reached during the thermophilic phase of the process. At this stage, most pathogens are inactivated, and the resulting composted biomass emits fewer pollutants.

Nevertheless, significant challenges remain, particularly regarding the persistence of pharmaceuticals and the presence of both conventional and biodegradable plastic bags, which may affect composting dynamics. Thermophilic conditions are often insufficient to completely degrade these materials, and both micro- and macroplastic residues have been detected in composted matter, raising concerns about potential environmental contamination [29].

Currently, too few studies are available to confirm that composted dog feces can be safely applied as fertilizer in crop production. However, this material has been proposed for alternative uses, such as land reclamation in areas degraded by human activity [30]. For these reasons, the potential of dog feces as fertilizer remains limited, and its composting requires further research and dedicated technological solutions.

### 3.3. Environmental Impact of Dog Feces: Nitrogen Compound Emissions

#### 3.3.1. Ammonia and Nitrous Oxide Emissions

Composting manure significantly reduces emissions of carbon dioxide (CO_2_), methane (CH_4_), and nitrous oxide (N_2_O) into the atmosphere. Nitrogen fluxes from feces originate primarily from undigested protein, which is hydrolyzed by putrefactive microorganisms into peptides and then free amino acids. In the subsequent deamination step, ammonia (NH_3_) or ammonium ions (NH_4_^+^) are formed [31]. The latter, being plant-available, forms only under specific environmental conditions. Gaseous ammonia emissions rise markedly at soil pH > 8 and temperatures above 20 °C [32].

Conditions particularly favorable for ammonia release from feces-contaminated soils occur in Southern Europe (e.g., Italy, the Iberian Peninsula). In contrast, soils in Northern and Central Europe are generally more acidified. This acidification is undesirable in agriculture, where lime fertilizers are commonly applied to increase soil pH [33]. Both ammonia and ammonium ions undergo further transformations within the nitrogen cycle—specifically through nitrification, producing nitrites (NO_2_^−^) and nitrates (NO_3_^−^). This process is a key pathway for nitrous oxide emissions, as N_2_O is a potent greenhouse gas (GHG) with a global warming potential (GWP) 273 times greater than that of carbon dioxide [34].

Global emissions of nitrous oxide (N_2_O) and ammonia (NH_3_) from dog and cat excreta are estimated at approximately 43 Gg N_2_O–N yr^−1^ and 654–864 Gg NH_3_–N yr^−1^, respectively [2].

It should be noted that these estimates refer only to emissions from pet food production and do not include nitrous oxide emissions originating from dog excreta (Table 1).

#### 3.3.2. NO_x_ and VOCs—Underestimated Sources of Urban Emissions?

Beyond GHGs, dog feces may also contribute to local air pollutant emissions, particularly nitrogen oxides (NO_x_) and volatile organic compounds (VOCs). In addition to ammonia and nitrous oxide, the decomposition of organic matter, including composting, can also release nitrogen oxides—nitric oxide (NO) and nitrogen dioxide (NO_2_)—collectively referred to as NO_x_. Although they account for only about 0.1% of total nitrogen compound emissions, NO_x_ plays a significant role in determining air quality [37].

There is a notable lack of literature directly addressing NO_x_ emissions from dog feces, as such sources are typically excluded from emission inventories. Nevertheless, these emissions may be particularly relevant in industrialized and densely populated urban areas [38]. Indeed, it is in these settings that dog feces are most abundant and where air pollution issues are especially severe.

When discussing the atmospheric impacts of nitrogen oxides, volatile organic compounds (VOCs) should also be considered. These include, among others, ketones, alcohols, terpenes, and various other carbon-based compounds (excluding methane, which is typically analyzed separately due to its role in global warming) [39]. Together with NO_x_, VOCs drive the formation of photochemical smog in cities and contribute to the synthesis of tropospheric ozone—a compound toxic to humans, animals, and plants [40].

To date, no studies have quantified the contribution of dog feces to VOC emissions. Despite their chemical similarity to livestock manure, dog feces remain largely overlooked in environmental pollution assessments, and their potential role in climate change has been virtually absent from public discourse. The emissions from such waste are localized, primarily affecting urban green areas where feces are often left on lawns. In this way, organic matter enters the environment in an uncontrolled manner, and the true scale of this phenomenon remains poorly understood [41].

### 3.4. Phosphorus—The Underestimated Component of Dog Feces and Its Environmental Consequences

In discussions of the environmental impact of companion animals, attention is usually directed first toward resource use and greenhouse gas emissions. Phosphorus—a key biogenic element—is often given less attention, even though dog excreta, both feces and urine, serve as important sources of this nutrient in the environment. While soil contamination from feces can be partly controlled through collection and disposal, there is no such control over urine, which infiltrates the soil directly. Moreover, even in the limited number of studies that address the environmental effects of dog urine, the focus is usually on nitrogen—its influence on vegetation, soil acidification, and microbiome diversity—whereas the role of phosphorus is almost entirely overlooked [42,43].

Phosphorus is essential for plant growth and is most frequently discussed in the context of agricultural overfertilization, where its excess is leached from fields into surface waters, causing eutrophication and severe disruption of aquatic ecosystems [44]. In a study by Hobbie et al. [45] on nutrient pollution in urban runoff entering the Mississippi River, researchers estimated the contribution of dogs and other domestic animals to nitrogen and phosphorus loads. Strikingly, 76% of phosphorus originating from household waste was attributed to pet excreta. Analyses further revealed that approximately half of the phosphorus excreted by dogs is released via urine, making its control particularly challenging. Unlike feces, which can be collected (at least in principle), urine is typically discharged directly onto urban surfaces, resulting in uncontrolled phosphorus dispersion into the environment.

It is also worth noting that, according to the same study, about 40% of dog feces are not collected by owners and degrade in situ [46]. On average, dog feces contribute around 5 kg P per hectare annually in urban soils. Phosphorus can remain bound in soils for centuries due to its low mobility. Phosphorus-rich soils favor ruderal plant species with rapid growth rates, which tend to outcompete less competitive native flora. The resulting decline in plant biodiversity cascades through the trophic web, disrupting populations of dependent pollinators, birds, and small mammals [14].

### 3.5. Overlooked Emission Pathways in Companion Animal Systems: Packaging, Plastics, and Hygiene Products

While gaseous emissions remain the most studied aspect of environmental impact, waste streams linked to packaging and synthetic materials represent additional, often overlooked contributors to the footprint of dog ownership. Caring for dogs extends far beyond meeting their basic physiological needs. Many owners also invest in providing physical activity, mental stimulation, and overall comfort [47]. At first glance, daily routines such as walking, playing, and feeding may seem unrelated to environmental pollution. However, these seemingly harmless activities generate substantial secondary waste—pet food packaging, waste bags, broken leashes, toys and bedding made of synthetic materials, and used grooming accessories—all of which may degrade into microplastics.

Despite this, scientific literature provides little quantitative data on waste generated by dog ownership beyond excreta and fecal collection bags. It is estimated that dog waste bags alone account for approximately 0.6% of global plastic pollution, equivalent to 0.7–1.23 million tons annually [48]. Considering the scale of the pet food and accessories market [4], the total amount of waste associated with dog care is likely to be considerably higher.

Dog owners’ consumer choices also influence the environmental footprint of pet care. The market now offers not only conventional plastic waste bags but also biodegradable alternatives. Advances in polymer technology have enabled the production of fully biodegradable plant-based materials that match the durability and performance of traditional plastics [49]. Under suitable composting conditions, some biodegradable bags can degrade completely within 90 days [50]. However, instances have been reported where bags labeled as “compostable” failed to decompose during testing, suggesting that they contained petroleum-derived components and may thus act as additional sources of microplastic pollution. Consequently, despite good intentions, dog owners may inadvertently contribute to environmental contamination [51].

Another significant aspect involves emissions associated with pet food packaging. The growing number of companion animals has driven rising demand for dog food, treats, and diverse packaging formats. Many producers still rely on non-biodegradable, non-compostable materials, which are linked to volatile organic compound (VOC) emissions during their production and disposal. The European dog food market alone accounted for over 40% of global market value in 2024, and packaging demand has increased proportionally. VOC emissions from the production of biodegradable polymers such as polylactic acid (PLA) or polybutylene adipate terephthalate (PBAT) are relatively low, although the addition of plasticizers and dyes can increase them. The highest VOC release occurs during the thermal sealing of packaged food, when compounds such as formaldehyde and ethyl acetate may be emitted. Nevertheless, all packaging materials must comply with European regulatory standards [52].

In summary, the European Union continues to implement increasingly advanced environmental regulations that encompass packaging for companion animal products. The Packaging and Packaging Waste Regulation (PPWR), adopted in 2024, prohibits oxo-degradable plastics, restricts single-use packaging, and requires packaging design to prioritize reuse and recyclability [53]. The EU thus remains a global leader in environmental legislation [54]. However, in many regions, the absence of equivalent regulations perpetuates the environmental burden associated with companion animal waste. On a global scale, emissions and pollution stemming from packaging, waste bags, and accessories remain a significant yet often overlooked component of the environmental pawprint.

### 3.6. Strategies for Reducing Emissions from Dog Excreta

The simplest way to mitigate emissions from dog excreta is to provide nutritionally balanced diets tailored to the animal’s body weight, age, and activity level. This is particularly relevant for amino acids, as any excess is either metabolized or excreted. Dog owners can also actively reduce the environmental footprint of their pets by selecting diets based on ingredients with lower GHG footprints. For example, poultry meat has a considerably smaller environmental burden than that of beef or lamb [55].

In response to growing demand for sustainable solutions, the pet food industry is increasingly exploring alternative protein sources with reduced environmental impacts. In particular, insect-based proteins—derived from black soldier fly larvae, mealworms, or crickets—are gaining popularity [56]. The Association of American Feed Control Officials (AAFCO) (2020. Champaign, IL: Official Publication. AAFCO) allows for the inclusion of plant-based by-products in companion animal diets [57]. However, as emphasized by Reilly et al. [58], the formulation of nutritionally complete and balanced dog foods from plant-based ingredients often requires supplementation with exogenous amino acids. Triggs et al. [59] also reported that dog owners tend to be more accepting of insect-based pet foods than of those formulated solely from plant ingredients.

To date, no studies have directly investigated gaseous emissions from dog feces. Data from other monogastric animals, such as pigs, where emissions and manure management have been extensively studied [60], may offer useful insights. Several approaches appear relevant:

- Dietary functional additives, including probiotic bacteria (e.g., Bacillus, Lactobacillus), prebiotics, symbiotic formulations, enzymes (e.g., proteases), plant extracts (e.g., piperine), and mixtures of organic acids [61].
- Proper manure management, such as timely removal from housing environments and adequate storage conditions. Additives like biochar have also been tested to reduce emissions from stockpiled manure [62].

However, physiological differences between dogs and livestock species must be taken into account before extrapolating these findings. Composting dog feces also represents a promising approach. He et al. [63] observed that adding nitrites (NO_2_^−^) to composted livestock manure significantly increased N_2_O emissions, showing a strong correlation between nitrite concentration and gas release. Notably, N_2_O emissions typically begin later in the composting process, once easily degradable carbon sources such as sugars are depleted. Supplementing compost with fresh food waste delayed the main emission phase and reduced total N_2_O output by approximately 20%. This observation is especially relevant given the unfavorable C:N ratio of dog feces: excess nitrogen combined with limited carbon availability may enhance N_2_O emissions during composting.

Recent research has also emphasized the need to address the management of dog excreta—particularly urine—in urban environments. Allen et al. [64] demonstrated that dog urine can cause acute changes in soil chemistry in urban greenspaces, leading to elevated nitrogen concentrations and increased electrical conductivity in soils near commonly used walking paths and play areas. In their study, ammonium concentrations averaged 103.9 mg kg^−1^ near poles and trees, compared with only 6.7 mg kg^−1^ at distances of 8 m from these paths. These findings highlight that, in densely populated areas, uncontrolled deposition of dog urine and feces can locally alter nutrient cycles and soil properties, contributing not only to environmental degradation but also to social conflicts arising from insufficient waste management practices. Therefore, developing urban policies that promote proper waste collection and awareness campaigns among pet owners is crucial for mitigating these impacts.

Taken together, these findings illustrate that the environmental pawprint of dogs results from multiple interacting factors, including diet composition, waste management practices, and owner behavior. Analogies drawn from livestock research indicate that mitigation strategies developed for monogastric animals can be adapted to companion animals when carefully modified. Moreover, urban management measures—such as designated dog waste collection areas and public awareness programs—may further reduce emissions. Future research should focus on the quantitative assessment of these emissions and the development of evidence-based guidelines to support sustainable pet ownership.

## 4. Conclusions

The analysis of the environmental pawprint of dogs underscores the need to move beyond viewing them solely as individual household animals. In contrast to livestock, which has been systematically monitored for emissions for decades, dogs remain largely absent from such assessments despite their vast global population. Yet, in densely populated urban areas, emissions associated with dogs can reach levels comparable to those from small livestock farms.

Dog excreta—particularly urine—represent an uncontrolled and often underestimated source of environmental pollution, introducing nitrogen, phosphorus, and various organic compounds into soils and surface waters. From a social perspective, the prevailing owner mindset that “one dog makes little difference” collectively amplifies the environmental burden associated with pet ownership. Educational initiatives and locally implemented policies could therefore play a key role in mitigating these effects.

Detailed quantitative studies on emissions from dog excreta are urgently needed, as is the development of effective mitigation strategies—both dietary (e.g., feed composition adjustments, use of functional additives) and management-related (e.g., composting optimization, emission-reducing amendments). Solutions adapted from livestock production, when properly tailored to the context of companion animals, could prove valuable. In parallel, coordinated public policies and owner education campaigns would enhance awareness and promote more sustainable practices.

In the long term, harmonizing legal frameworks and incorporating dogs into national and international emission inventories may become essential. Such integration would enable more accurate quantification of their environmental impact and support the development of evidence-based mitigation strategies.

## Figures and Tables

**Table 1:** Global N_2_O emissions from livestock and companion animals (dogs) in CO_2_-equivalents per year, and per animal for fur-bearing species (minks).

Species	Share of N_2_O (%)	N_2_O (Tg CO_2_-eq yr^−1^)	Reference
cattle	12.90	494.05	[35]
poultry	20.20	115.59	[35]
pig	18.00	152.22	[35]
American mink *	47.00	13.16 kg	[36]
dog **	10.19	64.00 ± 16	[5]

* Values estimated per animal. ** For dogs, N_2_O share includes only emissions related to dog food production; the value (64 ± 16 Mt CO_2_-eq yr^−1^) represents combined CH_4_ and N_2_O emissions according to the cited reference.

## Data Availability

The data presented in this study are available on request from the corresponding author due to restrictions.
